# *Borrelia burgdorferi* Complement Regulator-Acquiring Surface Protein 2 Does Not Contribute to Complement Resistance or Host Infectivity

**DOI:** 10.1371/journal.pone.0003010

**Published:** 2008-08-20

**Authors:** Adam S. Coleman, Xiuli Yang, Manish Kumar, Xinyue Zhang, Kamoltip Promnares, Deborah Shroder, Melisha R. Kenedy, John F. Anderson, Darrin R. Akins, Utpal Pal

**Affiliations:** 1 Department of Veterinary Medicine, University of Maryland, College Park, Maryland, United States of America; 2 Department of Microbiology and Immunology, University of Oklahoma Health Sciences Center, Oklahoma City, Oklahoma, United States of America; 3 Department of Entomology, Connecticut Agricultural Experiment Station, New Haven, Connecticut, United States of America; Walter and Eliza Hall Institute of Medical Research, Australia

## Abstract

*Borrelia burgdorferi*, the pathogen of Lyme disease, cycles in nature through *Ixodes* ticks and mammalian hosts. At least five Complement Regulator-Acquiring Surface Proteins (BbCRASPs) are produced by *B. burgdorferi*, which are thought to assist spirochetes in host immune evasion. Recent studies established that BbCRASP-2 is preferentially expressed in mammals, and elicits robust antibody response in infected hosts, including humans. We show that *BbCRASP-2* is ubiquitously expressed in diverse murine tissues, but not in ticks, reinforcing a role of BbCRASP-2 in conferring *B. burgdorferi* defense against persistent host immune threats, such as complement. BbCRASP-2 immunization, however, fails to protect mice from *B. burgdorferi* infection and does not modify disease, as reflected by the development of arthritis. An infectious *BbCRASP-2* mutant was generated, therefore, to examine the precise role of the gene product in spirochete infectivity. Similar to wild type *B. burgdorferi, BbCRASP-2* mutants remain insensitive to complement-mediated killing *in vitro*, retain full murine infectivity and induce arthritis. Quantitative RT-PCR assessment indicates that survivability of BbCRASP-2-deficient *B. burgdorferi* is not due to altered expression of other *BbCRASP*s. Together, these results suggest that the function of a selectively expressed *B. burgdorferi* gene, *BbCRASP-2*, is not essential for complement resistance or infectivity in the murine host.

## Introduction

*Borrelia burgdorferi* is the causative agent of Lyme disease, the most prevalent vector-borne disease in the United States and Europe [1], [2]. In nature, *B. burgdorferi* cycles between rodent reservoirs and *Ixodes scapularis* ticks. This complex enzootic life cycle requires successful colonization and coordinated transmission between strikingly different host and vector environments. It is thought that differential gene expression plays an important role in allowing the spirochete to navigate the transitions between hosts and in establishing persistent infection [3]–[6]. Due to the availability of excellent murine models of Lyme disease, *B. burgdorferi* gene expression through the tick-rodent transmission cycle can be examined in the laboratory [7]–[11]. These efforts may provide important clues for understanding functions of microbial gene products that support *B. burgdorferi* persistence in nature [3]–[6], [12], [13].

The genes encoding the Complement Regulator-Acquiring Surface Proteins (BbCRASPs) of *B. burgdorferi* are differentially expressed in the pathogen life cycle [14], [15]. As many as five BbCRASPs were identified that bind host proteins of the factor H (FH) family, and possibly contribute to the spirochete defense against host complement-mediating killing[16]–[18]. *BbCRASP-1* (also known as *cspA* or *bba68*) and *BbCRASP-2* (also called *cspZ* or *bbh06*), located on linear plasmids lp54 and lp28-3 respectively, share little sequence homology with other *BbCRASP* sequences. In contrast, *BbCRASP-3*, *-4* and *-5* are sequentially similar and belong to the *erp* paralog family and are known as *erpP* (*bbn38*), *erpC* and *erpA* (*bbp38* and *bbl39*), respectively. Collectively these *erp* genes are also known as *ospE*, and are encoded on multiple cp32 plasmids [19]–[22]. The gene *erpC* (located on cp32-2) and one of the three *erpA* genes (located on cp32-5) currently lack TIGR database annotations, as the sequenced B31 M1 isolate lost these plasmids. Of all the *BbCRASP* genes, *BbCRASP-2* is the only gene without paralogous family members, and is therefore unique in *B. burgdorferi* [17].

Evasion of host complement is especially important for *B. burgdorferi*, as it establishes an extracellular and disseminated infection in many tissue environments where the complement system is readily available through host vasculature or body fluids. The complement system includes soluble membrane binding proteins which, upon contact with foreign cells, become activated, and are then capable of direct chemical lysis via membrane disruption [23], [24]. Specific regulatory proteins, such as FH family proteins, protect the host from self-inflicted damage by preventing unwarranted complement activation. Pathogens such as *Candida albicans*, *Neisseria meningiditis*, and *Streptococcus pneumonia* have been shown to bind host FH, and that FH binding in *N. gonorrhea* and *B. burgdorferi* provides protection against complement killing *in vitro* [25]–[29]. BbCRASPs have been identified according to their ability to bind proteins of the FH family, although individual BbCRASPs vary in their affinities for particular FH family proteins. For example, only BbCRASP-1 and -2 preferentially bind factor H-like protein (FHL-1), while BbCRASP-3, -4 and -5 selectively bind factor H-related protein (FHR-1) [17], [30], [31]. BbCRASPs also vary in their interaction with uncharacterized serum proteins [14], [32]. Though the binding affinities and the expression profiles of the BbCRASPs have been studied, the independent role of each BbCRASP in *B. burgdorferi* infectivity is not clear. Recently studies using a non-infectious mutant demonstrated that the loss of BbCRASP-1 sensitized the *B. burgdorferi* to complement-mediated lysis in human serum, an effect that can be rescued with gene complementation [25]. While there is some disagreement as to the expression of BbCRASP-1 during mammalian infection, RT-PCR analysis indicate that it is only expressed transiently at the tick bite site and in ticks [14], but not expressed in mice [33]. BbCRASP-1 therefore, may not play an essential role in mammalian infection [33], but could be important in spirochete survival in feeding ticks. Although the above set of studies suggest an important role for BbCRASPs in spirochete immune evasion, the precise role of individual BbCRASPs, or their orchestrated role in the *B. burgdorferi* infection cycle is not clear, largely because infectious BbCRASP-deficient *B. burgdorferi* have not yet been successfully generated [14].

*BbCRASP-2* is expressed by *B. burgdorferi* during murine infection [14], [34], and infected hosts, including human patients, readily generate BbCRASP-2-specific antibodies [17], [35]. This protein is conserved among *B. burgdorferi* isolates [22], reported to be localized on the spirochete surface [17] and has recently been suggested as a possible target for a second generation Lyme disease vaccine [17], [35]. The previous studies also suggest a possible functional role for BbCRASP-2 in immune evasion and pathogen survival [17], [22]. In order to test this hypothesis, we sought to determine whether BbCRASP-2 is consistently produced in diverse murine tissues throughout the infection, and whether BbCRASP-2 immunization could provide host immunity and influence disease outcome. To explore the precise role of BbCRASP-2 in *B. burgdorferi* infectivity of a mammalian host, we assessed how targeted deletion of *BbCRASP-2* in an infectious isolate influences *B. burgdorferi* infection in the murine model of Lyme borreliosis. Functional characterization of microbial ligands that are differentially expressed in the complex enzootic cycle of *B. burgdorferi* is critical to understanding the adaptive strategies of a pathogen that has evolved to persist in diverse tissue environments resulting in multi-system disorders.

## Materials and Methods

### Bacteria, Mice and Ticks

*Borrelia burgdorferi* infectious isolate A3 [36], a clonal derivative of B31 M1, was used throughout the study. Female C3H/HeN mice between 4 and 6 weeks old purchased from the National Cancer Institute. Mice were inoculated with a single subcutaneous injection of 10^5^ spirochetes per mouse. All animal procedures were in compliance with the guidelines set by the Institutional Animal Care and Use Committee. The ticks used in this study belong to a colony that is reared and maintained in the laboratory as described [37].

### PCR

Mice were sacrificed following infection, and the heart, tibiotarsal joint, and skin samples were removed and frozen in liquid nitrogen. *B. burgdorferi-*infected ticks were isolated by allowing ticks to feed on an infected murine host as described [37]. RNA was extracted using TRIzol reagent (Invitrogen) and further treated with DNase I (Invitrogen), and finally purified using the RNeasy kit (Qiagen). RNA was used as a template for reverse-transcriptase polymerase chain reaction (RT-PCR) using the AffinityScript cDNA synthesis kit (Stratagene). The primers used for PCR reactions are indicated in Supplementary Table S1. Quantitative PCR analysis was performed using iQ SybrGreen Supermix (BioRad) as previously described [13]. For quantitative analysis of gene expression, the target transcripts were normalized to the number of *flaB* transcripts, whereas for quantitative measurement of *B. burgdorferi* burden in infected tissues, *flaB* transcripts were normalized to mouse or tick *β-actin* levels. All quantitative PCR results were checked for specificity by melting curve analysis.

### Production of recombinant BbCRASP-2 protein and BbCRASP-2 antibodies

Recombinant BbCRASP-2 protein was produced in *E. coli* using the pET303/CT-His Champion vector (Invitrogen) using specific primers (Supplementary Table S1). Recombinant BbCRASP-2 was fused with a C-terminal 6-histidine tag for purification, and lacked the peptides encoding the lipidation signal. Polyclonal antibodies against recombinant BbCRASP-2 were generated in mice as described earlier [37], [38]. Briefly, recombinant BbCRASP-2 (10 μg/animal) was emulsified in complete Freund's adjuvant (Sigma) and injected into groups of 10 mice. The animals were boosted twice at 10 days intervals with the same dose of antigen in incomplete Freund's adjuvant (Sigma) and the sera were collected two weeks following the second boost. Titer and specificity of the serum was tested by ELISA and immunoblot blot as described previously [39].

### Proteinase K accessibility assay

Proteinase K accessibility assays were performed as described [40]. Briefly, *B. burgdorferi* (2×10^8^) were gently washed three times in 1 ml of PBS (pH 7.4) and collected by centrifugation at 4,000×g for 4 min. Washed spirochetes were then gently resuspended in 1 ml of PBS and split into two equal 500 μl volumes. One aliquot received 200 μg of proteinase K (PK) (Sigma) while the other aliquot received an equal volume of PBS without PK. Both aliquots were incubated for 1 h at room temperature before the addition of 10 μl of phenylmethylsulfonylfluoride (Sigma) to stop PK activity. Spirochete suspensions were subsequently pelleted by centrifugation at 10,000×g for 10 min and resuspended in PBS for immunoblot analysis using antibodies against BbCRASP-2, FlaB, or OspA.

### Active immunization and infection studies

Groups of mice (6 animals/group) were immunized with adjuvant containing either recombinant BbCRASP-2, or adjuvant containing the same volume of phosphate buffered saline (PBS) in similar fashion as describe in above paragraph. Ten days after the final boost, mice were infected with a subcutaneous injection of *B. burgdorferi* (10^5^ spirochetes/mouse). Mice were sacrificed after 7, 12, 15 and 30 days of infection. Heart, tibiotarsal joint, and skin samples were collected and frozen in liquid nitrogen. RNA was isolated from infected tissues and *B. burgdorferi* burden was measured using quantitative PCR. *B. burgdorferi*-infected mice were examined for swelling and histology of the tibiotarsal joints as detailed [13], [41], [42].

### Generation and phenotypic analysis of *BbCRASP-2* mutant

BbCRASP-2-deficient *B. burgdorferi* was generated by homologous recombination replacing the entire open reading frame of the *BbCRASP-2* gene with a kanamycin resistance cassette [13], [38], [39], [43], [44] using primers as indicated in Supplementary Table S1. DNA fragments flanking the *BbCRASP-2* open reading frame on the 5′ and 3′ sides were PCR-amplified and inserted into the plasmid pXLF10601. This plasmid was sequenced to confirm identity and electroporated into wild-type *B. burgdorferi* A3, and transformants were selected for growth in the presence of kanamycin. PCR analysis was used to confirm the intended recombination event using primers P5-P12. Immunoblotting analysis using BbCRASP-2 antibodies was performed to confirm the loss of BbCRASP-2. The plasmid profile of the mutant *B. burgdorferi* was assessed to confirm no loss of wild type plasmids [13], [38], [44].

The serum sensitivity of the *BbCRASP-2* mutant was measured using a published procedure [16]. Briefly, triplicate samples of wild type *B. burgdorferi* or isogenic *BbCRASP-2* and *BbCRASP-1* mutant [25] were seeded into 1 ml tubes at a final concentration of 5×10^6^ bacteria per ml. These aliquots were incubated in either 50% normal human serum or 50% heat-inactivated human serum. At 0, 1, 4, and 16 hours after the addition of serum, spirochete viability was examined using dark-field microscopy. Normal human serum (Sigma) collected from healthy, anonymous donors with no reactivity to *B. burgdorferi* after chemiluminescent immunoblot analyses were used in the assay.

To examine the phenotype of the *BbCRASP-2* mutant *in vivo*, the mutant and wild type *B. burgdorferi* were separately inoculated into 2 groups of mice (6 animals/group, 10^5^ spirochetes/mouse). A skin sample, the heart and the joints from infected mice were isolated at day 7, 12 and 18 after challenge, and *B. burgdorferi* burdens were measured by quantitative RT-PCR analysis. Before sacrifice, ear and spleen tissues from the mice were cultured in BSK medium for the presence of viable *B. burgdorferi.* Rear ankle joints of individual mice were measured before infection and at 7, 12 and 18 days after infection until sacrifice. Acquisition of wild type and mutant *B. burgdorferi* by nymphal *I. scapularis* ticks was performed as described earlier [37]. Briefly, groups of C3H mice (6 animals/group) were infected with wild type or *BbCRASP-2* mutant *B. burgdorferi* (10^5^ spirochetes/mouse). Following 12 days of infection, 25 *I. scapularis* nymphs were placed on each mouse. The ticks were forcibly detached from the mice following repletion and immediately stored in liquid nitrogen. *B. burgdorferi* burdens in each tick sample were measured as described earlier by quantitative RT-PCR analysis.

### Statistical analysis

Results are expressed as the mean ± standard error of the mean (SEM). The significance of the difference between the mean values of the groups was evaluated by two-tailed Student *t* test.

## Results

### *BbCRASP-2* is ubiquitously expressed during murine infection

**Figure 1:**
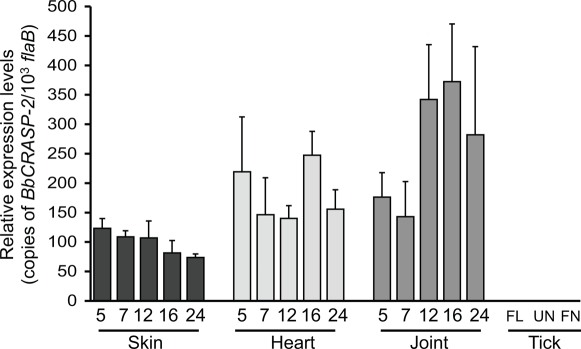
Ubiquitous expression of *BbCRASP-2* in infected mice. The relative expression levels of *BbCRASP-2* in the murine host, and in representative life stages of ticks, are analyzed and presented as copies of *BbCRASP-2* transcript per 1000 copies of *flaB* transcripts. Total RNA was isolated from multiple tissues of *B. burgdorferi*-infected mice (6 mice/group) at day 5, 7, 12, 16 and 24 following challenge, from infected fed larva (FL) after 3 days of feeding, unfed nymphs following larval molting (UN) and fed nymphs (FN) after 3 days of feeding. *BbCRASP-2* transcripts were measured using quantitative RT-PCR. Error bars represent the mean ± SEM from four quantitative PCR analyses of two independent murine infection experiments. *BbCRASP-2* transcripts were abundant in all murine tissues tested but were not detected in any stages of the ticks.

To understand role of BbCRASP-2 in *B. burgdorferi* infectivity, we first assessed the transcript levels of *BbCRASP-2* in multiple murine tissue locations where *B. burgdorferi* persists during infection, and in various stages of infected ticks. C3H mice were infected with *B. burgdorferi*, and heart, joints and skin samples were collected at days 5, 7, 12, 16, and 24 following infection. Larval and nymphal ticks were fed on parallel groups of 15 day-infected mice (25 ticks/mouse) and fully engorged ticks were isolated following 3 days of repletion. Batches of infected fed larvae were allowed to molt and then collected as unfed nymphs. Total RNA was prepared from murine and tick samples, and subjected to quantitative RT-PCR to measure the *B. burgdorferi BbCRASP-2* transcript levels. *BbCRASP-2* is ubiquitously and consistently expressed throughout infection (Fig. 1A), but was undetectable in larval or nymphal ticks. As reported earlier [17], the infected mice developed a specific antibody response against BbCRASP-2 (data not shown).

### BbCRASP-2 immunization failed to evoke protective immunity in mice

**Figure 2:**
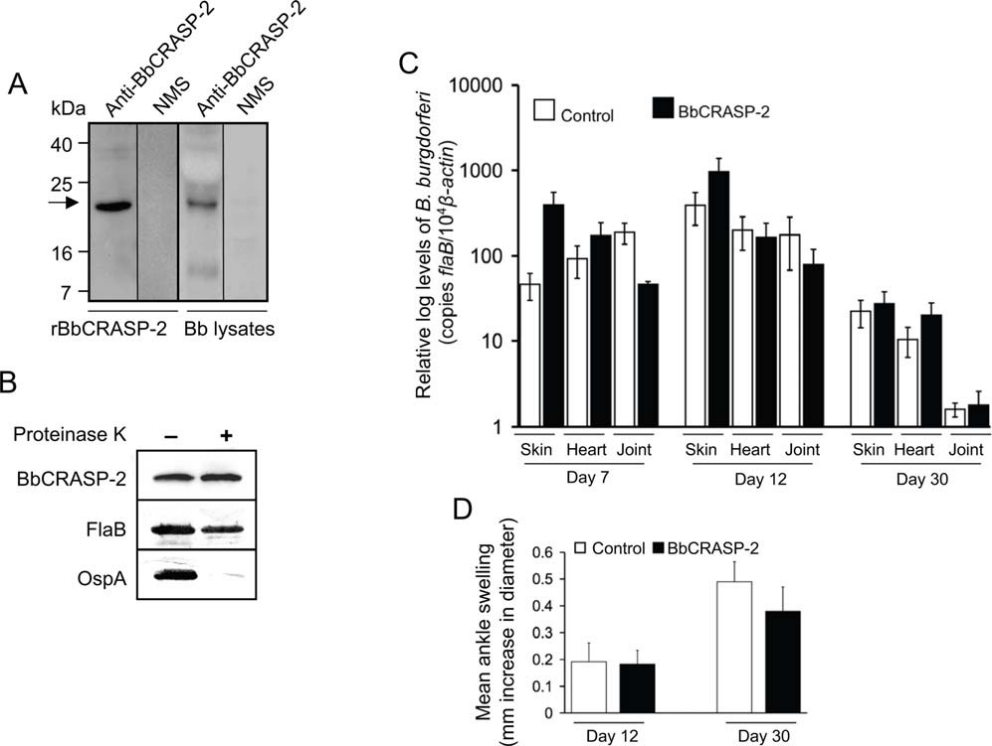
BbCRASP-2 immunization does not interfere with *B. burgdorferi* infectivity. *A*, Recognition of recombinant and native BbCRASP-2 proteins by immunized murine serum, as assessed by immunoblotting. Recombinant BbCRASP-2 protein (50 ng) or *B. burgdorferi* lysates (200 ng) were probed with BbCRASP-2 antiserum or normal mouse serum (NMS). The arrow indicates the position of BbCRASP-2. Migration of protein standards is shown to the left. *B*, BbCRASP-2 is not sensitive to proteinase K-mediated degradation of *B. burgdorferi* surface proteins. Viable spirochetes (2×10^8^ cells) were incubated with (+) or without (−) proteinase K for removal of protease sensitive surface proteins and processed for immunoblot analysis using BbCRASP-2 antibodies. *B. burgdorferi* OspA and FlaB antibodies were utilized as controls for surface-exposed and sub-surface proteins, respectively. *C*, Comparable levels of *B. burgdorferi* in rBbCRASP-2-immunized or control mice. Groups of mice (6 animals/group) were immunized with either rBbCRASP-2 or PBS (control) mixed with adjuvant, and 10 days after final immunization mice were infected with *B. burgdorferi* (10^5^ spirochetes/mouse). The spirochete burdens in both groups of mice were assessed by measuring copies of the *B. burgdorferi flaB* gene at 7, 12 and 30 days following infection. Amounts of murine *ß-actin* were determined in each sample and used to normalize the quantities of spirochete RNA. Bars represent the mean measurements ± SEM from four quantitative PCR analyses from two independent infection experiments. Levels of *B. burgdorferi* were similar in BbCRASP-2-immunized (black bars) and control mice (white bars). The burdens found in BbCRASP-2-immunized mice were not statistically significant from the control burdens in any tissue or time point (*P*>0.05, n = 4). *D*, Severity of joint swelling in BbCRASP-2-immunized and infected mice. Groups of mice (6 animals/group) were immunized with BbCRASP-2 (black bars) or PBS (control, white bars) and infected with *B. burgdorferi*. Development of joint swelling was assessed after 12 and 30 days of spirochete challenge by measuring the largest diameter of the rear tibiotarsal joints using a digital caliper.

Because BbCRASP-2 is surface-exposed [17], immunogenic, and expressed throughout the murine infection, we next assessed if immunization of mice using recombinant BbCRASP-2 could elicit protective immunity and influence the outcome of Lyme disease. To accomplish this, we produced recombinant BbCRASP-2 in *E. coli* and immunized the murine host with purified BbCRASP-2. Separate groups of C3H mice (6 animals/group) were immunized with either BbCRASP-2 or PBS (control) mixed with similar volume of adjuvant. ELISA (data not shown) and immunoblotting performed after final boosting indicated that immunized mice had developed strong antibody titer that specifically recognized recombinant and native BbCRASP-2 (Fig. 2A). Although previous immunofluorescence studies indicated that BbCRASP-2 antibodies recognized native antigen on the surface of the intact spirochetes [17], our proteinase K accessibility assay indicated that BbCRASP-2 is not significantly exposed on the spirochete surface (Fig. 2B). Ten days after the final immunization mice were needle-inoculated with *B. burgdorferi* (10^5^ spirochetes/mouse). *B. burgdorferi* levels were measured by quantitative PCR from skin, heart and joint samples collected at 7, 12, 15 and 30 days after infection. Results indicated no significant difference in spirochete burden between mice immunized with BbCRASP-2 or the control at any time points (Fig. 2C). Quantitative RT-PCR analysis further showed no difference between the transcript levels of *BbCRASP-2* in immunized and control groups (data not shown). These results indicated that BbCRASP-2 immunization did not influence the ability of *B. burgdorferi* to establish infection. Development of ankle swelling (Fig. 2D) or histopathological changes in the joint tissue (data not shown) in *B. burgdorferi*-infected mice immunized with BbCRASP-2 did not differ from the control, suggesting that host BbCRASP-2 antibodies fail to influence the ability of *B. burgdorferi* to induce arthritis in the murine host.

### Generation of an infectious isolate of BbCRASP-2-deficient *B. burgdorferi*

**Figure 3:**
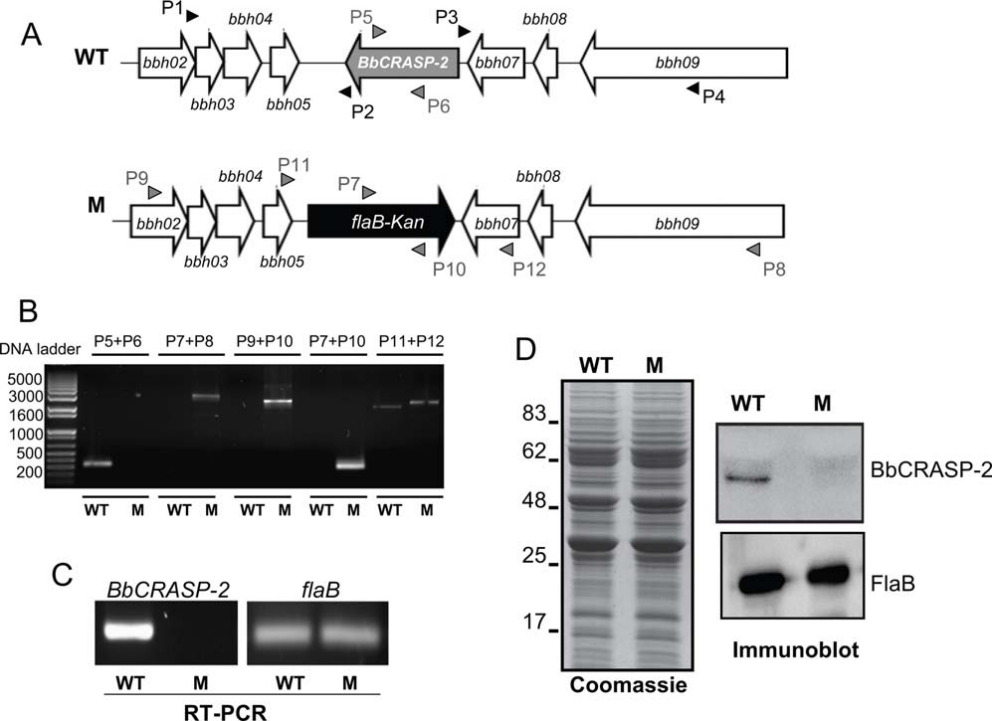
Construction and analysis of the *BbCRASP-2* mutant *B. burgdorferi*. *A*, Schematic drawings of the wild type isolate (WT) and the *BbCRASP-2* mutant (M) at the *BbCRASP-2* (*bbh06*) locus. Genes *bbh02*, *bbh03*, *bbh04*, *bbh05*, *bbh07*, *bbh08* and *bbh09* (white box arrows) and the kanamycin-resistance cassette driven by the *B. burgdorferi flaB* promoter (*flaB-Kan*, black box arrow) are indicated. Primers P1-P4 (black arrow-heads) amplified 5′ and 3′ arms for homologous recombination, regions flanking up- and down-stream of the *BbCRASP-2* locus and ligated on either side of the *flaB-Kan* cassette as detailed in the text. *B*, Integration of the mutagenic construct, *flab-Kan*, in the intended genomic locus. Primers 5–12 (gray arrow-heads, positions indicated in Fig. 3A) were used for PCR analysis using isolated DNA from wild type (WT) or *BbCRASP-2* mutant *B. burgdorferi* (M) and subjected to gel electrophoresis. The combination of primers used for PCR is indicated at the top. Migration of DNA ladder is shown on the left. *C*, RT-PCR analysis of *BbCRASP-2* transcripts. Total RNA was isolated from either the wild type (WT) or *BbCRASP-2* mutant (M) *B. burgdorferi*, converted to cDNA and used to PCR-amplify regions within *BbCRASP-2* and *flaB*, and these amplicons visualized on a gel. *D*, Protein analysis of wild type (WT) or *BbCRASP-2* mutant (M) *B. burgdorferi*. Equal amounts of protein from wild type or *BbCRASP-2* mutant spirochetes were separated on an SDS-PAGE gel, and either stained with coomassie blue (left panel) or transferred onto a nitrocellulose membrane and probed with BbCRASP-2 or FlaB antibodies. Migration of protein standards is shown to the left in kDa.

Since BbCRASP-2 immunization did not influence spirochete infection, we created a BbCRASP-2-deficient *B. burgdorferi* to more directly assess the role of the gene product in *B. burgdorferi* survival and infectivity. An infectious isogenic mutant was created by replacing the *BbCRASP-2* open reading frame with a kanamycin resistance cassette via homologous recombination (Fig. 3A). PCR analysis was performed to ensure that the antibiotic cassette was appropriately inserted into the intended chromosomal locus (Fig. 3B), and that the plasmid profile of the mutant was unchanged. Out of 4 transformed clones that grew in antibiotic-containing media, 2 clones contained the desired integration of the antibiotic cassette and retained the same set of plasmid as in the parental isolate. One of the mutant clones was chosen for further study. RT-PCR analysis showed that *BbCRASP-2* mRNA was absent in the mutant (Fig. 3C), and that *BbCRASP-2* mutagenesis did not impose polar effects on the transcription of surrounding genes, *bbh05* and *bbh07* (data not shown). The *BbCRASP-2* mutant spirochetes displayed a similar protein profile to that of the wild type (Fig. 3D, left panel), except that the *BbCRASP-2* mutant failed to produce BbCRASP-2 protein (Fig. 3D, upper right panel).

### BbCRASP-2-deficiency did not affected serum resistance of *B. burgdorferi in vitro*

**Figure 4:**
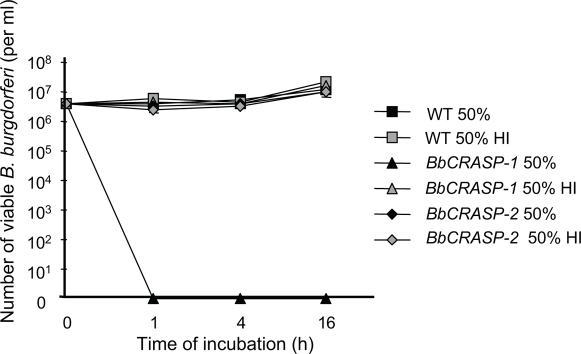
*BbCRASP-2* mutant *B. burgdorferi* is resistant to complement-mediated killing *in vitro*. Triplicate wells of spirochetes (5×10^6^ cells/ml) were exposed to 50% normal human serum or 50% heat-inactivated human serum (HI) and the viability of *B. burgdorferi* was assessed using dark-field microscopy. An isogenic *B. burgdorferi BbCRASP-1* mutant served as a serum-sensitive positive control, which were killed within 1 hour of incubation with complement active serum as expected (*P*<0.05, compared to corresponding HI control), while both wild type and *BbCRASP-2* remained fully resistant to serum (*P*>0.05).

*B. burgdorferi* is known to be resistant to complement-mediated lysis in serum, and deficiency of *BbCRASP-1* has been shown to render *B. burgdorferi* highly susceptible to serum-mediated killing *in vitro* [25]. Because *BbCRASP-2* is expressed by wild type spirochetes grown in culture, we assessed whether BbCRASP-2 deficiency affects the serum resistance of the spirochetes. Equal concentrations of wild type and *BbCRASP-2* mutant *B. burgdorferi* were separately incubated with human serum containing active complement and the serum sensitivity of each isolate was assessed. While the isogenic *BbCRASP-1* mutant were readily killed within 1 hour of serum exposure, the viability of the *BbCRASP-2* mutant did not differ significantly from that of the wild type (Fig. 4) indicating that BbCRASP-2 is not essential for *B. burgdorferi* resistance to complement-mediated lysis in serum.

### BbCRASP-2-deficient *B. burgdorferi* retain full murine infectivity

**Figure 5:**
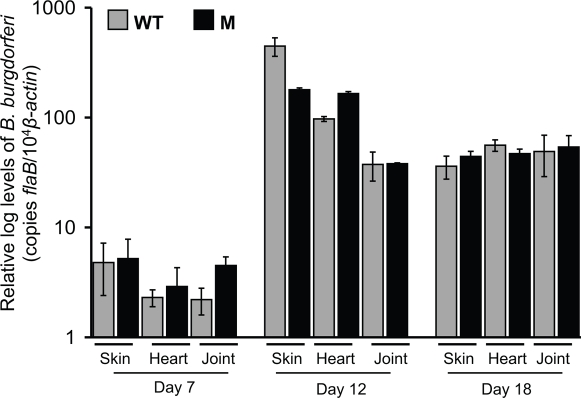
*BbCRASP-2* mutant *B. burgdorferi* retain full pathogenicity in the murine host. The *B. burgdorferi* burdens in multiple tissues of the infected mice are shown. Mice (6 animals/group) were infected with either the wild type or the *BbCRASP-2* mutant *B. burgdorferi* and spirochete burdens were analyzed as before by measuring copies of *B. burgdorferi flaB* RNA at day 7, 12 and 18 following infection. Amounts of murine *ß-actin* were determined in each sample and used to normalize the quantities of spirochete RNA. Bars represent the mean measurements ± SEM from four quantitative PCR measurements from two independent infection experiments. No difference between wild type and *BbCRASP-2* mutant levels was statistically significant in any tissue or time point measured (*P*>0.05, n = 4).

To examine whether the lack of BbCRASP-2 influences *B. burgdorferi* infectivity in a mammalian host, C3H mice were infected with wild type or *BbCRASP-2* mutant *B. burgdorferi*. Both the mutant and wild type spirochetes were readily cultured from ear and spleen tissues taken from mice 12 days after the inoculum (data not shown). When nymphal ticks were allowed to feed on infected mice, *BbCRASP-2* mutant *B. burgdorferi* were able to migrate into fed ticks at a similar level to the wild type spirochetes (data not shown). Quantitative RT-PCR further showed that the *BbCRASP-2* mutant established infection in mice in comparable levels to the parental isolate. No significant differences in the burdens of *BbCRASP-2* mutant and wild type isolates were detected in murine skin, heart and joint samples isolated after 7, 12 and 18 days of infection (Fig. 5). Development of swelling in the murine joints infected with either the *BbCRASP-2* mutant or the wild type *B. burgdorferi* was also similar (data not shown). Overall, these results suggest that BbCRASP-2 is not essential for establishment of *B. burgdorferi* infection in the mouse model of Lyme disease.

### Loss of BbCRASP-2 function is not compensated by augmented or new expression of other BbCRASPs

**Figure 6:**
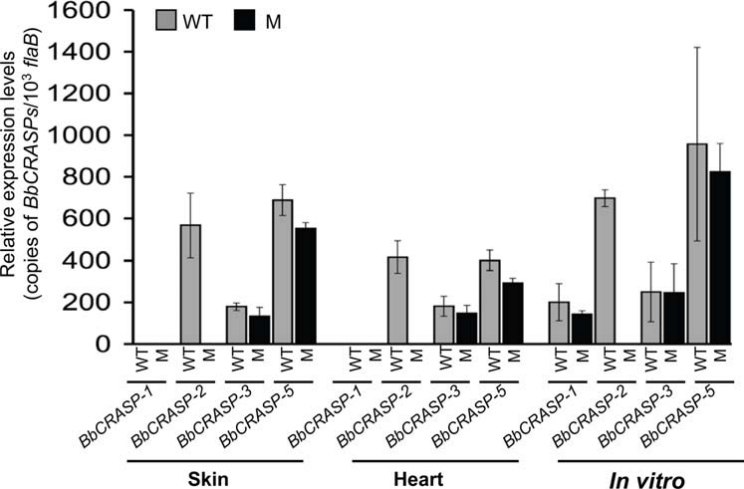
*BbCRASP-2* mutant *B. burgdorferi* express other *BbCRASP* genes at similar levels to the wild type spirochetes. The relative expression levels of *BbCRASP-1*, *-2*, *-3* and -5 were examined in the *BbCRASP-2* mutant and wild type spirochetes *in vitro* and *in vivo* by quantitative RT-PCR, and are represented as copies of gene per 1000 copies of *flaB* transcripts. Total RNA was isolated from *B. burgdorferi* isolates grown in culture (10^7^/ml), as well as multiple tissues of *B. burgdorferi*-infected mice following 12 days of infection. The experiments were replicated thrice, and bars represent the mean measurements ± SEM from four representative quantitative PCR measurements. Differences between *BbCRASP* transcripts in the wild type and mutant were not statistically significant (*P*>0.05, n = 4).

BbCRASP-2 deficiency did not affect the ability of the *BbCRASP-2* mutant to survive complement-mediated lysis *in vitro* or establish infection in a mammalian host *in vivo*. Since *B. burgdorferi* encodes multiple BbCRASPs that are capable of binding host complement regulators, we explored the possibility that the loss of *BbCRASP-2* could be compensated by altered expression of other potential *BbCRASP* genes, such as *BbCRASP-1*, *-3* and *-5*. We did not examine the expression of *BbCRASP-4*, as the parental B. burgdorferi isolate A3[36] lacks the non-essential cp32-2 plasmid housing the gene. To examine *BbCRASP* expression, groups of 6 C3H mice were needle-inoculated with wild type or BbCRASP-2-deficient *B. burgdorferi* (10^5^ spirochetes/mouse). Infected murine skin and heart samples were isolated 7, 12 and 18 days after infection, and expression of each *BbCRASP* was measured by quantitative RT-PCR. *In vitro* expression of *BbCRASP* genes was also assessed by growing wild type and mutant *B. burgdorferi* in BSK medium to various cell densities (10^6^–10^8^ spirochetes/ml) and analyzed by quantitative RT-PCR. The expression profiles of *BbCRASP-1*, *-3*, and *-5* remained unaltered in the *BbCRASP-2* mutant when compared to the wild type spirochetes, both *in vitro* and *in vivo*, such as in the murine skin and heart tissues at all time points. *BbCRASP* expression in cultured spirochetes grown *in vitro* to a density of 10^7^/ml and in infected murine skin and heart tissue samples at 12 days of infection is presented (Fig. 6). These results suggest that the loss of BbCRASP-2 function is not compensated by alteration or new expression of other *BbCRASP* genes (Fig. 6).

## Discussion

*B. burgdorferi* express up to five BbCRASPs that are either structurally unique, such as BbCRASP-1 and -2, or closely related, BbCRASP-3, -4 and -5 [17], [18], [21], [45]. These BbCRASPs are differentially expressed and are postulated to confer defense against host-derived complements via specific interaction with FH family proteins [16], [17], [31], [32]. The precise role of individual BbCRASPs in the *B. burgdorferi* infection cycle, however, is currently unclear. BbCRASP-2 is specifically produced in the mammalian host including humans, and is immunogenic, and thus, is thought to be important in spirochete pathogenesis and may be useful in a future Lyme disease vaccine [17], [35]. Here, we show that BbCRASP-2 is ubiquitously expressed throughout murine infection, evoking a detectable antibody response. However, BbCRASP-2 immunization fails to protect the host against *B. burgdorferi* infection or influence the genesis of disease. Targeted deletion of BbCRASP-2 did not impair the ability of the mutants to resist serum-mediated killing *in vitro*, establish infectivity *in vivo*, or the severity of disease. Deficiency of *BbCRASP-2* expression in mutants is not functionally compensated by the enhanced expression of other *BbCRASP* genes. BbCRASP-2, therefore, is not essential for *B. burgdorferi* survivability *in vitro*, and based on the time periods covered in the present study, we conclude that BbCRASP-2 function is dispensable for *B. burgdorferi* infectivity mice and in feeding ticks.

Immunization of murine hosts against specific *B. burgdorferi* antigens, such as DbpA [46], [47], OspC [48] and OspA [49] can elicit production of borreliacidal antibodies, and thus confer protective host immunity possibly by killing spirochetes *in vivo* when administered prior to spirochete infection. In contrast, other *B. burgdorferi* antigens are also described, such as BmpA/B [13] and Arp [50], [51], that fail to protect host against *B. burgdorferi* infection, but influence pathogenesis either by reduction of the *B. burgdorferi* burden in selected tissues [13] or by modifying the disease without affecting spirochete load [50], [51]. The failure of BbCRASP-2-specific host immunity to influence *B. burgdorferi* pathogenesis indicates that BbCRASP-2 antibodies lack significant neutralizing effects on spirochetes, possibly due to the lack of significant surface-exposure of the antigen. This is further confirmed by the observation that the BbCRASP-2-deficient *B. burgdorferi* displayed no phenotypic defects in their ability to infect the murine host or induce disease. Nevertheless, as *BbCRASP-2* is abundantly produced by *B. burgdorferi* throughout infection and evokes development of specific antibody, our data reinforces an earlier contention that BbCRASP-2 could be a reliable marker for the serodiagnosis of Lyme disease [17], [35].

*B. burgdorferi* expresses select lipoproteins [19] in the mammalian host [52], including BbCRASP-2. Owing to its ubiquitous expression in the host and known affinity for FH family proteins [17], BbCRASP-2 appears to be important for *B. burgdorferi* protection against persistent host immune threats, such as complement system. BbCRASP-2 is conserved among infectious *B. burgdorferi* isolates, which also suggests an important role for this protein in the infectivity of *B. burgdorferi* [22]. The plasmid lp28-3 that houses BbCRASP-2 is retained in most of the *B. burgdorferi* clones isolated from experimentally infected hosts [53]–[56]. Previous studies attempting to identify specific plasmids required for *B. burgdorferi* infectivity indicate that lp28-3 plasmid may not be strictly necessary for spirochete infectivity [55] while other studies suggest that several plasmids, including lp28-3, in the correct combinations, may be required to mediate *B. burgdorferi* infection of the mammals [54]. Nevertheless, our data conclusively show that the function of BbCRASP-2 is not essential for *B. burgdorferi* survival against serum-mediated killing *in vitro* or host infectivity. The *BbCRASP-2* mutant fails to produce both *BbCRASP-1* and *BbCRASP-2* in the murine host, yet still retains full infectivity, which suggests that complement evasion in the host, if relevant, could be mediated by BbCRASP-3 and -5, which remain fully expressed by the mutant. Alternatively, binding to certain FH family proteins may not be essential to the spirochetes, as previous studies indicate that *B. burgdorferi* are able to infect and cause disease in FH-deficient mice [57].

In summary, we present direct evidence that *B. burgdorferi* adapts for the loss of a differentially expressed and abundant lipoprotein during mammalian infection. Past studies also identified additional *B. burgdorferi* genes encoding potential lipoproteins, such as BBA64 [58], [59] or OspD [60], [61] that display highly regulated expression *in vivo* but lack an essential role in *B. burgdorferi* persistence in tick-mouse infection cycle. Here we show that BbCRASP-2 function is also dispensable for infectivity of the murine hosts or in feeding ticks. Together, these results highlight *B. burgdorferi* as a unique pathogen that has evolved versatile adaptive strategies to survive and establish infection in a diverse array of host species, including humans.

## Supporting Information

Table S1 (0.05 MB DOC)
