# Supplementary material for: Borrelia burgdorferi Complement Regulator-Acquiring Surface Protein 2 Does Not Contribute to Complement Resistance or Host Infectivity
Source: PLoS One. 2008 Aug 20;3(8):e3010. doi: 10.1371/journal.pone.0003010 (PMC2526170; doi:10.1371/journal.pone.0003010)
Supplement: Table S1 — (0.05 MB DOC) [file pone.0003010.s001.doc]

| **Sequence (5’ to 3’)** | **Purpose** |
| --- | --- |
| TTCAATCAGGTAACGGCACA | Forward primer for *B. burgdorferi flaB* Quantitative RT-PCR (qRT-PCR). |
| GACGCTTGAGACCCTGAAAG | Reverse primer for *B. burgdorferi* *flaB* qRT-PCR. |
| AGAGGGAAATCGTGCGTGAC | Forward primer for mouse *β-actin* qRT-PCR. |
| CAATAGTGATGACCTGGCCGT | Reverse primer for mouse *β-actin* qRT-PCR. |
| TGCCAGACATGTTGCTGATT | Forward primer for *BbCRASP-2* qRT-PCR. |
| CCCCCTCAAGTTCTACAGCA | Reverse primer for *BbCRASP-2* qRT-PCR. |
| TT*TCTAGA*ATGGATGTTAGTAGATTAAATCAGA | Forward primer for producing recombinant BbCRASP-2. The italicized *XbaI* site is included for cloning. |
| ATA*CTCGAG*TAATAAAGTTTGCTTAATAGCT | Reverse primer for producing recombinant BbCRASP-2. The italicized *XhoI* site is included for cloning. |
| AGAGAGAG*GCGGCCGC*AAACAACAAAGACTCGCATATTCA | Primer P1, used to amplify and clone the 5' flanking region to generate the *BbCRASP-2* mutant. The italicized *NotI* site is included for cloning purposes. |
| GGA*CTGCAG*GTTTCAATCTCTCTAAACATG | Primer P2, used to amplify and clone the 5' flanking region to generate the *BbCRASP-2* mutant. The italicized *PstI* site is included for cloning purposes. |
| AGGA*CTCGAG*TTCATAGCCTCTAGCACAAG | Primer P3, used to amplify and clone the 3' flanking region to generate the *BbCRASP-2* mutant. The italicized *XhoI* site is included for cloning purposes. |
| AGG*GGTACC*ATTTTGGAGTAGGGCTAAA | Primer P4, used to amplify and clone the 3' flanking region to generate the *BbCRASP-2* mutant. The italicized *KpnI* site is included for cloning purposes. |
| TGCCAGACATGTTGCTGATT | Primer P5, forward primer used to confirm the deletion of *BbCRASP-2*. |
| CCCCCTCAAGTTCTACAGCA | Primer P6, reverse primer used to confirm the deletion of *BbCRASP-2*. |
| GGTTGCATTCGATTCCTGTT | Primer P7, forward primer used to confirm the deletion of *BbCRASP-2*. |
| TGGAAATCCTCCATGGGAAA | Primer P8, reverse primer used to confirm the deletion of *BbCRASP-2*. |
| AATTCAATATGGAGGAAGTTTTAGGGCT | Primer P9, forward primer used to confirm the deletion of *BbCRASP-2*. |
| ATTCCGACTCGTCCAACATC | Primer P10, reverse primer used to confirm the deletion of *BbCRASP-2*. |
| TAAATGTATATGCTCCAGTAAACTAAACCAC | Primer P11, forward primer used to confirm the deletion of *BbCRASP-2*. |
| CCTCAGGATGTCATATTGGTAGCA | Primer P12, reverse primer used to confirm the deletion of *BbCRASP-2*. |
| TCTTATTTACACACTAAACAACATTGACC | Forward primer for *B. burgdorferi* *bbh05* qRT-PCR. |
| TAAACGCACGGCGTATAAAG | Reverse primer for *B. burgdorferi* *bbh05* qRT-PCR. |
| TGATGAATTTTTGGGAATGTTTT | Forward primer for *B. burgdorferi* *bbh07* qRT-PCR. |
| TGAATTTTATACATGGGTACAGTTTTG | Reverse primer for *B. burgdorferi* *bbh07* qRT-PCR. |
| CTAAAAGCAATTGGTAAGGAACTG | Forward primer for *BbCRASP-1* for qRT-PCR |
| TCAATAAGATCGTAAGGACCAACT | Reverse primer for *BbCRASP-1* for qRT-PCR |
| GTTTAAACGCTGGGGGACAT | Forward primer for *BbCRASP-3* for qRT-PCR. |
| ACGCAATATGTTCAGCACCA | Reverse primer for *BbCRASP-3* for qRT-PCR. |
| TGATGAGCAAAGTAGTGGT | Forward primer for *BbCRASP-5* for qRT-PCR. |
| GAATGTCCCCCAGCGTTTA | Reverse primer for *BbCRASP-5* for qRT-PCR. |

**Table S1**: Oligonucleotide primers used in the study.
